# Tipping elements in the human intestinal ecosystem

**DOI:** 10.1038/ncomms5344

**Published:** 2014-07-08

**Authors:** Leo Lahti, Jarkko Salojärvi, Anne Salonen, Marten Scheffer, Willem M. de Vos

**Affiliations:** 1 Department of Veterinary Biosciences, University of Helsinki, PO Box 66, FI-00014 Helsinki, Finland; 2 Laboratory of Microbiology, Wageningen University, PO Box 8033, 6700 EJ Wageningen, The Netherlands; 3 Department of Bacteriology and Immunology, Immunobiology Research Program, Haartman Institute, University of Helsinki, PO Box 21, FI-00014 Helsinki, Finland; 4 Aquatic Ecology, Wageningen University, PO Box 47, 6700 AA Wageningen, The Netherlands; 5 These authors contributed equally to this work

## Abstract

The microbial communities living in the human intestine can have profound impact on our well-being and health. However, we have limited understanding of the mechanisms that control this complex ecosystem. Here, based on a deep phylogenetic analysis of the intestinal microbiota in a thousand western adults, we identify groups of bacteria that exhibit robust bistable abundance distributions. These bacteria are either abundant or nearly absent in most individuals, and exhibit decreased temporal stability at the intermediate abundance range. The abundances of these bimodally distributed bacteria vary independently, and their abundance distributions are not affected by short-term dietary interventions. However, their contrasting alternative states are associated with host factors such as ageing and overweight. We propose that the bistable groups reflect tipping elements of the intestinal microbiota, whose critical transitions may have profound health implications and diagnostic potential.

## Abstract (web summary)

Intestinal microbes can have important effects on our health. Here, the authors analyse the gut microbiota composition in 1,000 western adults and find that certain bacteria are either abundant or nearly absent, and that these alternative states are associated with ageing and overweight.

## Graphical abstract

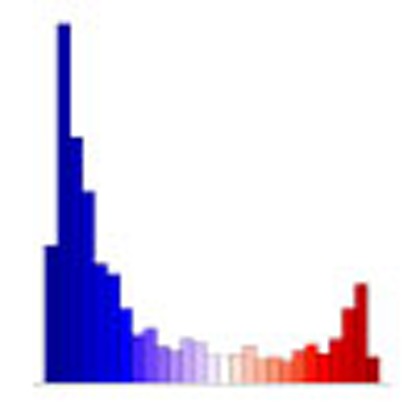

The versatile and dynamic microbial ecosystems inhabiting our intestinal tract can have profound impact on well-being and health^1^,^2^,^3^,^4^. Although the compositional and functional properties of the intestinal microbiota have been studied extensively, we have limited understanding of the mechanisms that control this complex ecosystem^5^,^6^. A fundamental question is whether the overwhelming variability of the microbiota composition occurs along smooth phylogenetic gradients or through abrupt switches between alternative stable states^7^. In ecology, the concept of alternative stable states separated by tipping points has emerged as a theoretical framework with major practical implications^8^.

Here we demonstrate the same properties in the human intestinal ecosystem. While previous studies have focused on analysing ecosystem-wide configurations in human intestinal microbiota^7^,^9^,^10^, our analysis of a thousand western adults shows that there are distinct groups of bimodally distributed bacteria that tend to be either very abundant or nearly absent, and exhibit reduced stability at the intermediate abundance range between these two contrasting states. We propose that the bistable groups we identified represent ‘tipping elements’^11^, specific components of the intestinal microbiota that exhibit alternative stable states linked to the overall ecosystem state and our physiology. While further studies are needed to establish the possible causal relations, the bistable taxa can serve as indicators of the community state and its potential health associations, and resetting these ‘bacterial switches’ may be a radically new way to approach the rapidly growing number of health issues related to the intestinal microbiota, changing the way we look at management of the intestinal ecosystem.

## Results

### Identification of alternative states

**Figure 1:**
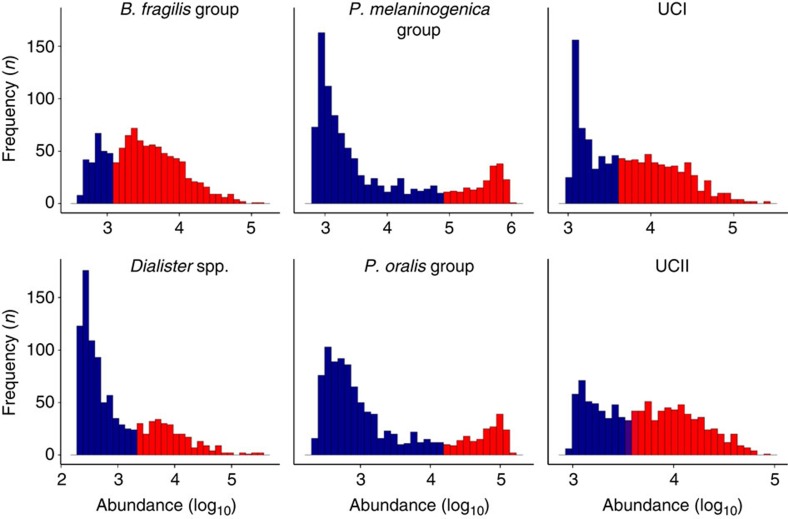
The bimodal bacteria. Logarithmic abundance distributions of the six bimodal phylogenetic groups that exhibit robust alternative states of low (blue) and high (red) abundance across intestinal microbiota of 1,006 western adults. The UCI and UCII refer to the uncultured Clostridiales I and II, respectively. The frequency of the observations is shown as a function of the phylogenetic microarray log_10_ signal^12^.

**Figure 2:**
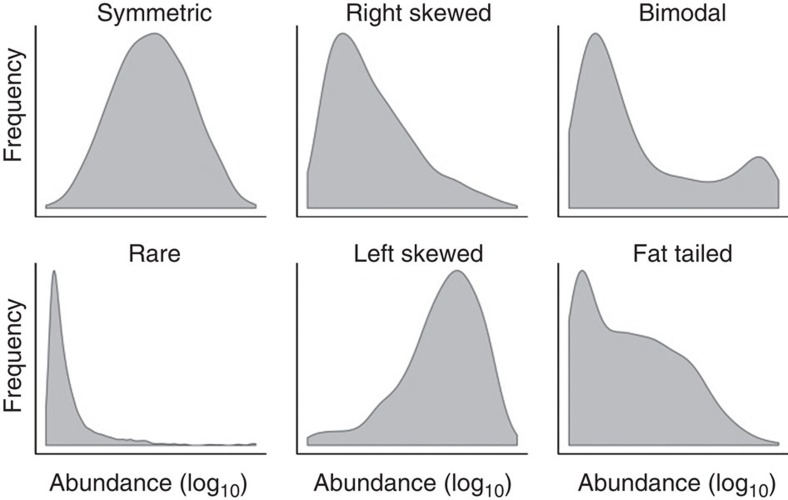
Bacterial abundance types. Bacterial abundance types include symmetric, skewed and bimodal logarithmic abundance distributions (Supplementary Table 1). The skewed types include prevalent left- and right-skewed types as well as rare bacteria. The bimodal types (Fig. 1) include cases with two distinct peaks, and cases with a peak at low abundance combined with a more widely varying fat tail of high-abundance subjects. The population frequencies of the log abundance across the 1,006 western adults are shown for representative examples from each category: *Anaerostipes caccae et rel.* (symmetric); *Serratia* spp. (rare); *Streptococcus bovis et rel.* (right skewed); *Faecalibacterium prausnitzii et rel.* (left skewed); *P. oralis et rel.* (bimodal distribution with two distinct peaks); and uncultured Clostridiales I (fat tailed, bimodal distribution with a variable high-abundance state).

To systematically investigate the hypothesis of alternative stable states in the intestinal microbiota, we analysed 130 genus-like phylogenetic groups that cover the majority of the known bacterial diversity of the human intestine^12^ (Supplementary Table 1). Our analysis is based on >2,000 standardized phylogenetic microarray hybridizations that represent the faecal microbiota of a thousand adults (*n*=1,006; 18–77 years) from 15 western countries (Europe and the United States; see Methods). On the basis of this extensive data collection, we identified several bacterial groups exhibiting robust bimodal abundance distributions (Fig. 1; Supplementary Table 2), including *Dialister* spp., relatives of *Bacteroides fragilis*, *Prevotella melaninogenica*, *P. oralis* and two groups of the uncultured Clostridiales (UCI and UCII; Supplementary Note 1). The strongly correlated *P. melaninogenica* and *P. oralis* were combined into a single *Prevotella* group (Supplementary Note 2). The other 124 genus-like groups that we investigated were either rare or exhibited symmetric or skewed abundance types (Fig. 2; Supplementary Table 1).

To detect alternative states in the intestinal bacteria, we used potential analysis that was recently applied to study analogous state shifts in climatology and forest ecosystems^13^,^14^. This theoretical framework for state identification in stochastic dynamical systems is well suited for studying the remarkably variable intestinal microbiota. We used bootstrap analysis to ensure that the findings were robust to random sampling variations, and selected for further analysis the taxonomic groups that showed consistent evidence for multimodality with a bootstrap support of ≥98% for *Dialister* spp., *P. oralis*, *P. melaninogenica*, UCI and UCII groups, and 93% for the *B. fragilis* group. The evidence was the strongest for two alternative states in all these groups, and further supported by an alternative approach, the prediction strength index (PSI)^10^,^15^, which provided strong^10^ evidence of bimodality (PSI>0.90) for *Dialister* spp., UCI, UCII and relatives of *P. melaninogenica* and *P. oralis*, and a moderate support for the *B. fragilis* group (PSI>0.80).

To control the possibility that the bimodal distributions could be explained by diet or other independent environmental factors due to potentially unbalanced sampling, we confirmed the bimodality in distinct subsets of the data collection, controlled potential cross-hybridization effects between closely related taxa (Supplementary Note 2) and investigated the available host and environmental parameters (see Methods). In all cases, except the *B. fragilis* group, the bimodality was supported in independent sample sets analysed with different DNA extraction methods, including two sets of samples extracted with different combination of mechanical and chemical lysis steps (*N*=401 and *N*=287). Moreover, the 287 samples were all from central Europe, and thus expected to be less heterogeneous regarding the long-term dietary patterns compared with the entire data collection that included subjects from the United States and from 14 countries in Europe. Since long-term habitual diets are correlated with geographic regions, the verification of bimodality in two different data sets with different geographical distributions supports our conclusions by reducing the possibility that the observed bimodality is driven by country-specific dietary or other life-style differences within the study cohort. Although all DNA preparations were identically processed for the microarray analysis, the verification in independent subsets of the data collection further helps to exclude the possibility that the detected bimodality derives from technical variation in sample processing.

Analysis of an additional data set of 138 individuals from five dietary intervention trials indicated that the observed bimodality is robust against short-term dietary interventions as we did not observe significant population-level differences in the mean abundances between the baseline and post-intervention samples in the bimodal taxa in these studies (*P*>0.05; *t*-test; Benjamini–Hochberg correction; see Methods). Remarkably, the bimodal patterns were also indicated among the 46 placebo-treated control subjects in these controlled dietary intervention trials (Supplementary Fig. 1), further supporting the conclusion that the population-level bimodal patterns and the observed state shifts cannot be explained by short-term dietary differences. Whereas the observed states were robust to short-term dietary interventions, this does not exclude the possibility that targeted dietary interventions could be potentially used to manipulate specific bistable groups. On the basis of the available data collected in intervention trials, a pronounced dietary effect on the bimodal taxa was observed, for instance, on a formula-based weight-loss diet during which 29% (9/31) of the subjects exhibited a switch from the high to the low-abundance state of *Dialister* spp. These profound individual changes were, however, masked by remarkable within-state variation among the other subjects so that the average group-level fold change following the intervention was moderate and only borderline significant (log_10_FC=−0.39; *P*=0.08; paired Wilcoxon test). Although the observed states appear predominantly robust to dietary variation, we and others have shown that the abundance of the implicated bacteria can be to some extent affected by specific interventions as the quantity and quality of consumed carbohydrates can alter the abundance of *Bacteroides* and *Prevotella* species^16^,^17^,^18^ as well as that of *Dialister* spp.^19^ However, the reported changes were relatively small (average |log_10_FC|<0.5) in the bimodal groups that we now report^16^,^17^ and thus induced mainly within-state variation according to the present analysis. Hence, further studies are needed to assess the possibility to induce state switches in bistable taxa based on dietary or other interventions.

### Temporal dynamics

**Figure 3:**
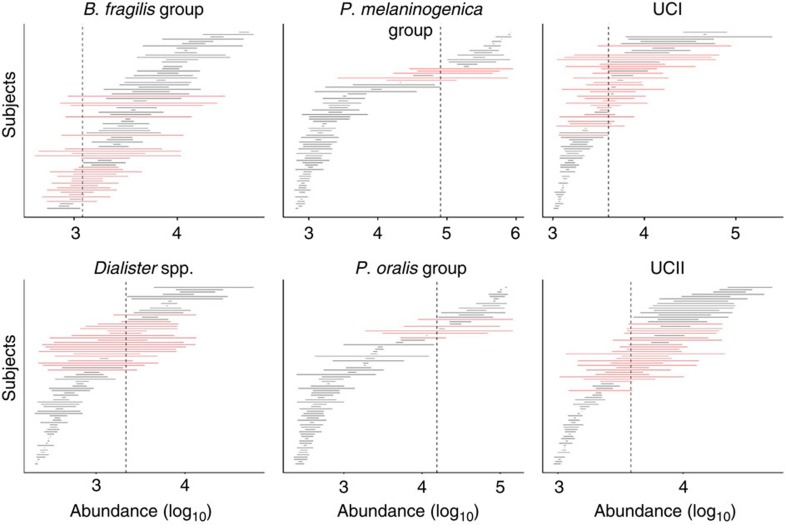
Temporal variation. Temporal variation in each bimodal group during the follow-up interval (1–9 months from the baseline for 95% of the subjects, see Methods). Each horizontal line indicates the abundance range of the indicated bacteria for a single subject. The 78 subjects (horizontal lines) are ordered based on their mean abundance. The red lines highlight subjects exhibiting a state shift (Supplementary Fig. 3) across the estimated tipping point (dashed vertical lines). The differences in the follow-up times did not significantly affect the observed fluctuations (*P*>0.05; Pearson correlation; Benjamini–Hochberg adjustment).

**Figure 4:**
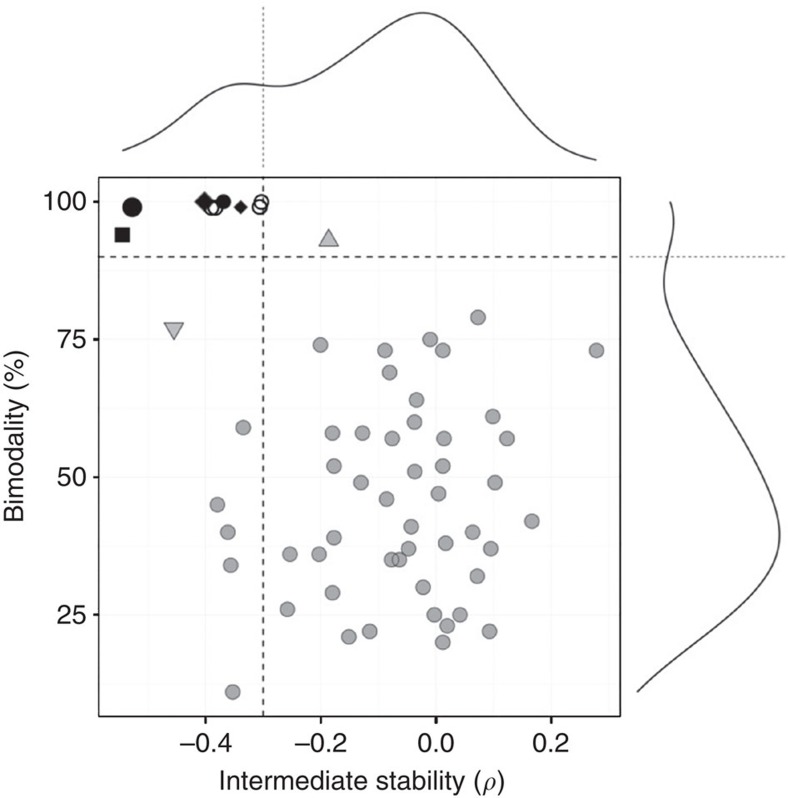
Bimodality is associated with reduced intermediate stability. Analysis of the 60 prevalent taxa indicates a group of bistable bacteria with robust bimodal abundance distributions (>90% bootstrap support; vertical axis) and reduced intermediate stability (*ρ*<−0.3; horizontal axis), as also indicated by the corresponding frequency histograms (side panels). The bimodality scores are shown for the 401 samples extracted by mechanical lysis^25^; the stability estimates are based on the 78 follow-up subjects. The black symbols indicate the bistable groups whose bimodality was supported in multiple data sets, including the *Dialister* spp. (square), *P. melaninogenica* (large diamond), *P. oralis* (small diamond), UCI (small circle) and UCII (large circle) groups. These show also significantly reduced intermediate stability compared with the other prevalent groups (*ρ*<−0.3; *P*<0.002; hyper-geometric test). The other bistable taxa (white circles) whose bimodality we could not verify in the other data sets include Firmicutes (*Clostridium* spp. (*sensu stricto*), relatives of *C. colinum*, *Lactobacillus plantarum* and uncultured Mollicutes). The bimodal *B. fragilis* group (grey upward triangle) has only moderately reduced intermediate stability (*ρ*=−0.19). The *C. difficile* group (grey downward triangle) has the third lowest intermediate stability.

The theory of stochastic dynamical systems predicts^8^ that alternative stable states are separated by an intermediate unstable region, called a tipping point, where even small fluctuations may lead to an abrupt, self-propagating shift to an alternative state. While this can give rise to bimodal abundance distributions^8^, as in our reported bimodal bacteria that tend to be either rare or abundant in most subjects, the observed bimodality could also be caused by other factors such as diet or yet unknown host factors, dividing the study cohort into two distinct subpopulations. Thus, bimodality of states provides only indirect evidence for bistability. In bistable systems, observations in the intermediate region between the alternative states may correspond to subjects in a transitional phase between the states^8^, for instance, due to changes in diet or physiological state. We therefore looked into the temporal dynamics of the states based on a subset of 78 subjects (27–63 years) in our main cohort with additional follow-up samples within 1–9 months from the baseline (see Methods). This complements our cross-sectional analysis that represents a static snapshot of the intestinal ecosystem in each individual. A closer look at the temporal fluctuations (Fig. 3) indicated that subjects closer to the tipping point indeed had larger temporal deviations, suggesting that the intermediate abundances appear less attractive than the contrasting alternative states. We confirmed this by quantifying the stability of bacterial abundances at the intermediate abundance range. The observed deviations from the baseline in the later time points were higher in subjects whose baseline abundances were closer to the intermediate (50%) abundance range, as indicated by significant negative correlation (Spearman’s coefficient *ρ*) between these variables for the *Dialister* spp. (−0.54), *P. melaninogenica* (−0.40), *P. oralis* (−0.34), UCI (−0.37), UCII (−0.53) and *B. fragilis* (−0.19) groups. These correlations were significant (*P*<0.01; upper tail (AS89) test; Benjamini–Hochberg adjustment) for all bimodal groups, except *B. fragilis* (*P*=0.1). Moreover, comparison between the altogether 60 prevalent taxa showed that the bimodal groups had significantly reduced intermediate stability compared with the other groups (Fig. 4; *P*<0.002; hyper-geometric test). This observation supports the hypothesis of two alternative stable states of low and high abundance divided by an unstable tipping point at the intermediate abundance range in these taxa, in line with the theoretical predictions outlined above for bistable systems^8^. Furthermore, the comparison between bimodality and intermediate stability in Fig. 4 suggested the presence of further bistable bacteria (Firmicutes), including *Clostridium* spp. (*sensu stricto*) and relatives of *C. colinum*, *Lactobacillus plantarum* and uncultured Mollicutes, as well as the *C. difficile* group that exhibits exceptionally low intermediate stability and a moderate support for bimodality, in line with earlier observations that a small fraction of healthy adults carry *C. difficile*^20^, one of the members of this group. Toxin-producing derivatives of *C. difficile* have been identified as the cause of severe and recurrent antibiotic-associated diarrhoea, and recent faecal transplantations have been shown to provide efficient means to manipulate its alternative states and treat the disease, resulting in complete recovery^21^,^22^. It is currently not known whether the alternative states of *C. difficile* as part of the normal microbiota are associated with the infection risk. While these observations suggest bistable properties for these taxa, we could not confirm their alternative states in the other tests. Furthermore, we analysed the temporal stability of the above-mentioned states, quantified as the fraction of subjects remaining in the initial state after a 3-month interval as estimated by Kaplan–Meier survival analysis (see Methods). The states with higher population frequencies were more stable in general (Supplementary Fig. 2a), in line with predictions based on the stationary-state continuous Markov process (Supplementary Fig. 3) and consistent with the idea that changes in species abundance distributions can be used to infer changes in the stability and resilience of alternative states^23^. The high-abundance *Prevotella* group was a notable exception as one of the least frequent but yet one of the most stable states. The alternative states of the reported bistable groups were also more stable than expected based on empirical distributions derived from the unimodal taxa (Supplementary Fig. 2b); the *Prevotella* group exhibited less state mixing than any other group (100%), followed by UCI (less mixing than in 93% of the comparison groups), *Dialister* spp. (83%) and UCII (78%). The *B. fragilis* group was again an exception (35%).

### Analysis of individual phylotypes and methanogenic archaea

Phylotype-level analysis of the six bimodal groups indicated that the observed bistability is often associated with particular phylotypes, and absent in others (Supplementary Figs 4 and 5; Supplementary Note 3). This could indicate potential antagonism or metabolic specialization between the implicated phylotypes. In certain groups, such as the UCI and UCII, the most prevalent phylotypes exhibited stronger bimodality than their higher-level genus-like group (Supplementary Fig. 4). Of the bistable groups indicated in Fig. 4, only the *Dialister* spp., relatives of *P. melaninogenica* and *P. oralis*, and UCI/UCII groups, whose bistability was systematically supported in the different analyses, had prevalent phylotypes according to our criteria. Moreover, we observed notable variation within the genus *Prevotella* as *P. ruminicola* exhibited only moderate correlation with the combined *P. oralis*/*P. melaninogenica* group (*r*=0.39). The most strikingly bimodal bacteria were uncultured phylotypes, as in the case of the *P. melaninogenica* and *P. oralis* groups, where the type species were detected only at low abundance, but the related uncultured bacteria exhibited bimodal distributions (Supplementary Fig. 4). *D. invisus* (*Dialister* spp.) is an example of a cultured species with a strong bimodal abundance distribution (Supplementary Fig. 5).

**Figure 5:**
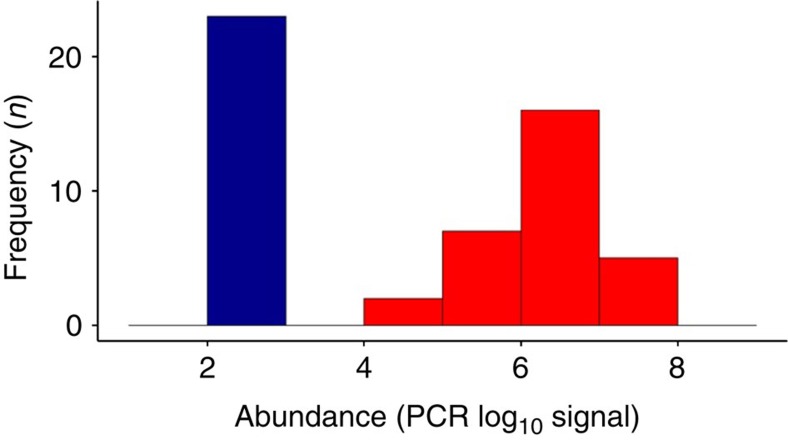
Abundance distribution of *Methanobrevibacter*, the dominant methanogenic archaeon in the human intestine. Quantitative PCR-based (qPCR)^25^ logarithmic abundance distribution, expressed as 16S rRNA gene copies per gram of faeces, indicates bimodality of this genus. *Methanobrevibacterium* spp. were detected in 47% of the 53 unique subjects from our main cohort for which the qPCR data were available. For subjects where *Methanobrevibacterium* spp. were not detected, the abundance is set at the detection limit of the assay. The colours indicate the two groups of subjects where the *Methanobrevibacter* spp. are absent or below the detection limit (blue), or detected (red).

We augmented the phylotype analysis with quantification of methanogenic archaea as this group is not targeted by the Human Intestinal Tract Chip (HITChip) phylogenetic microarray. The analysis of alternative states in methanogenic archaea is of interest as the current evidence is inconclusive regarding their prevalence in human populations. Traditionally, only a fraction of individuals have been considered as carriers, but a recent molecular study reported that the dominant archaeon, *Methanobrevibacter smithii*, is present in >95% of individuals^24^. We observed indications of bimodality in *Methanobrevibacter* spp. based on the quantitative PCR-based^25^ enumeration, which was available for 53 subjects from our main cohort. On the basis of this analysis, we found 47% of these unique subjects to be carriers of *Methanobrevibacter.* Moreover, the logarithmic abundance distribution (Fig. 5) exhibited clear bimodality, representing carriers and non-carriers. Quantitative PCR-based data for these archaea were available for 452 additional samples from 189 unique subjects, of which 57% were found to be carriers. Among the 53 subjects in common with our main cohort, the abundance of these archaea had moderate positive correlation with the *Prevotella* group (*r*=0.32; *P*=0.02; *t*-statistics), UCI (*r*=0.28; *P*=0.04) and UCII (*r*=0.23; *P*=0.1) groups that we also found to be associated with increased community diversity and metagenomic richness (Supplementary Fig. 6; Supplementary Note 4).

### Tipping elements and the ecosystem state

**Figure 6:**
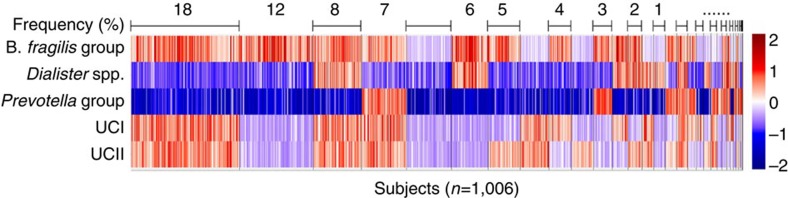
The tipping elements co-occur in various combinations. The bimodal groups (rows) co-occur in various combinations within the study population. Shading indicates the relative abundance (log_10_) for each group with respect to the identified tipping point between the alternative states of low (blue) and high (red) abundance. The 1,006 subjects are ordered based on the combination frequencies (top row). The most frequent combination (18%) corresponds to the high-abundance state of the UCI, UCII and *B. fragilis* groups combined with the low-abundance state of the *Dialister* spp. and *Prevotella* groups.

**Figure 7:**
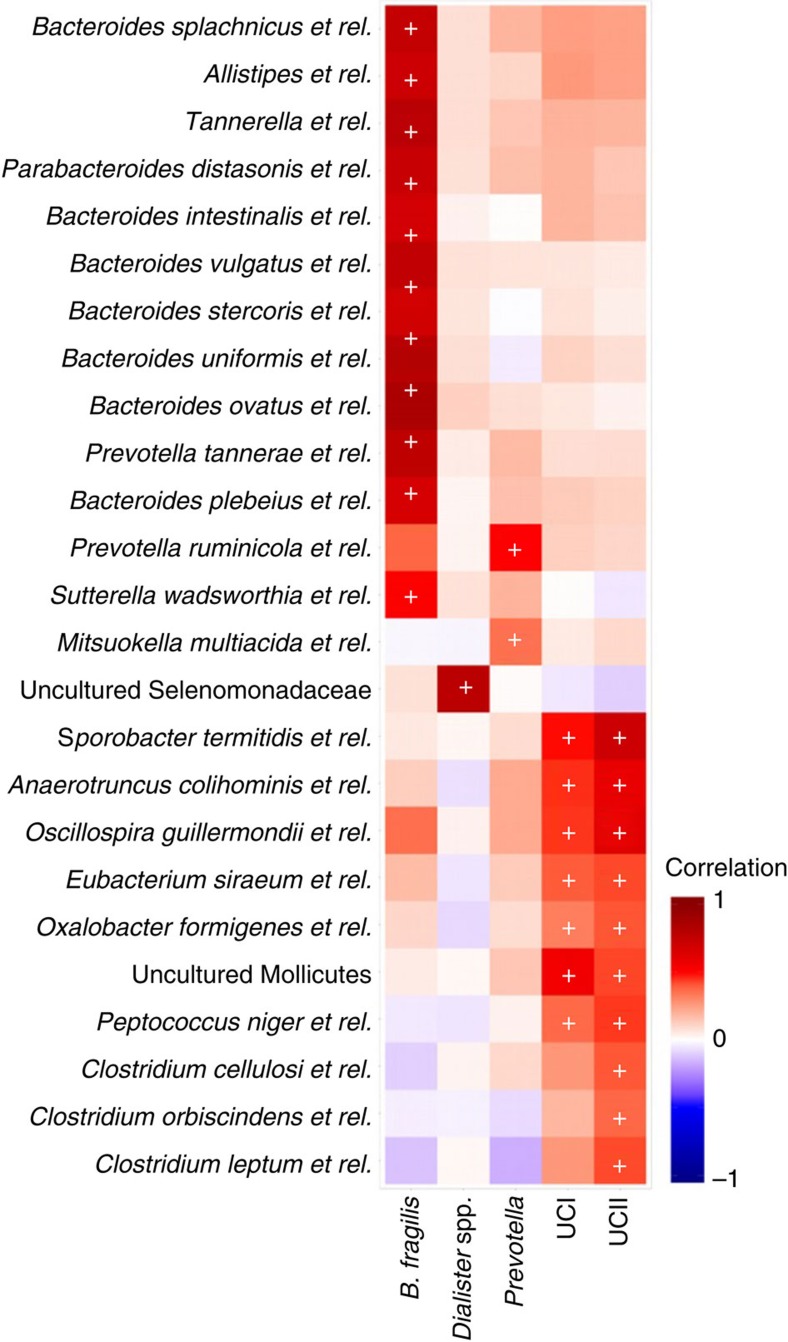
Covariation between the bimodal groups and other taxa. Summary of correlations between the bimodal groups (columns) and the 124 other genus-like groups targeted by the HITChip microarray; significant correlations with 25 groups (rows; *P*<0.05; |*r*|>0.25; Pearson correlation) were consistently observed in independent sample sets analysed using two different DNA extraction methods (mechanical lysis; *n*=401 and enzymatic lysis-based method; *n*=287), and in 100 bootstrap data sets sampled from the overall data collection (*n*=1,006). The remaining 99 groups that did not show significant correlations have been removed for clarity. The 32 correlations that were found to be consistently significant in all tests are indicated by ‘+’. Shading indicates Pearson correlation between the taxa across the 1,006 western adults.

While multimodality in specific microbial groups, notably the *Prevotella* genus, has previously been reported^10^, our analysis complements and extends these earlier studies by providing increased phylogenetic depth and sample size, and by suggesting previously unreported taxonomic groups exhibiting bistable properties that associate to the overall intestinal ecosystem state and host health. The six bimodal genus-like groups that we identified exhibited only weak preferences for mutual co-occurrence or exclusion (|*ρ*|<0.16 among the 401 samples with mechanical lysis; Fig. 6), except a positive correlation between UCI and UCII (*ρ*=0.50) and between *P. melaninogenica* and *P. oralis* (ρ=0.97), which we have combined into a single *Prevotella* group. The low- and high-abundance states of the resulting five bimodal groups yield altogether 32 (2^5^) possible state combinations with varying population frequencies (Fig. 6), suggesting that the alternative stable states associated with distinct subcommunities often coexist in a host in different combinations. We observed, for instance, a substantial overlap between the *B. fragilis* and *Prevotella* groups, with no significant differences in *B. fragilis* abundance between the low- and high-abundance *Prevotella* subjects (log_10_FC<0.05; *t*-test; *P*>0.05). Similar results on the genus *Bacteroides* have been reported earlier^26^, in contrast with other studies that have reported a notable trade-off between *Bacteroides* and *Prevotella* spp^6^,^9^,^27^. The bistable groups also covaried with other bacteria (Fig. 7), suggesting that their state shifts can be associated with wider changes in the overall ecosystem composition, and could be potentially used as indicators of the overall community state. Establishing possible causal relations between the bistable groups and ecosystem composition will require further studies, however.

Ecosystem-level investigation based on principal component analysis (PCA; Supplementary Fig. 7) and the PSI^10^,^15^ indicated two alternative states in the overall microbiota composition, roughly corresponding to low and high *Prevotella*, in line with previous reports^9^,^10^. The main principal component explained 16% of the overall variation in the data and exhibited a continuous gradient associated with *Bacteroides* spp. and relatives of *Oscillospira guillermondii* and *Faecalibacterium prausnitzii*. The second principal component exhibited a bimodal distribution associated with the *Prevotella* (relatives of *P. oralis* and *P. melaninogenica)* group, explaining 13% of the total variation. We did not observe robust bimodality on the other major principal components. A complementary analysis using the principal coordinates analysis with UniFrac distances^28^ yielded similar results, although the bimodality of the second (*Prevotella*) principal axis was less pronounced than in the standard PCA (Supplementary Fig. 7). Whereas the genus *Prevotella* and *Bacteroides* spp. have been earlier suggested as drivers of community-level enterotypes^9^, our observations extend and complement this analysis by identifying specific bacteria exhibiting bistable properties within these groups and analysing their temporal dynamics, and by demonstrating that these groups in fact frequently co-occur in a host in varying combinations. The high-abundance state of the *Prevotella* group had the most dominating effect on the ecosystem, with an average relative abundance of 10% among the subjects with the high-abundance state (8 and 2% for the co-occurring *P. melaninogenica* and *P. oralis* groups, respectively). Due to their lower relative abundances (≤2% in the high-abundance state) compared with the *Prevotella* group, the bimodal patterns in the *B. fragilis*, *Dialister* spp., UCI and UCII groups are more easily masked by variability in other, more smoothly varying taxa that correlate with these groups (Fig. 7), and hence the alternative states associated with these bacteria are easily overlooked in ecosystem-level analysis. Interestingly, whereas these less abundant groups had more moderate influence at the ecosystem-level variation, their high-abundance states were notably more prevalent compared with the high-abundance state of the more abundant *Prevotella* group, with the following population frequencies: *Prevotella* group (20%), *Dialister* spp. (28%), UCI (53%), UCII (57%) and *B. fragilis* (75%).

We also observed significant associations between the bistable bacteria, community diversity and the overall metagenomic gene richness (Wilcoxon test; *P*<0.01 for all groups, except *B. fragilis*; Supplementary Fig. 6; Supplementary Note 4) when we incorporated the recently reported metagenomic sequencing data that were available for 255 subjects^29^ in our main cohort. In our analysis of these meta-genomes, the high-abundance state of the *B. fragilis* and *Dialister* spp. groups were found to be associated with a low gene count, in line with their reported association with pronounced metabolic dysfunction^29^. On the contrary, we observed associations between the high-abundance state of the *Prevotella*, UCI and UCII groups and the high gene count, which has been linked to a potentially healthy metabolic phenotype^29^. The increased metagenomic richness could be explained by the increased community diversity associated with these taxa; among the bistable groups, the UCI and UCII showed the strongest association with the overall community diversity (*ρ*=0.5; *P*<0.001; upper tail (AS89) test). Whereas the community diversity exhibited also mild positive correlation with the *Prevotella* group (*ρ*=0.13; *P*=0.04), the overall community diversity was higher in the subjects with high-abundance UCI/UCII than in the subjects associated with high-abundance *Prevotella* (*P*<0.001; *t*-test). The higher diversity associated with the UCI/UCII groups (≤2% relative abundance) could be partially explained by the more dominating status of the high-abundance *Prevotella* group (10% relative abundance), which tends to reduce the overall community diversity. We did not observe significant associations between the *B. fragilis* and *Dialister* spp. groups and community diversity (|*ρ*|<0.07; *P*>0.3).

### Host factors and health status

**Figure 8:**
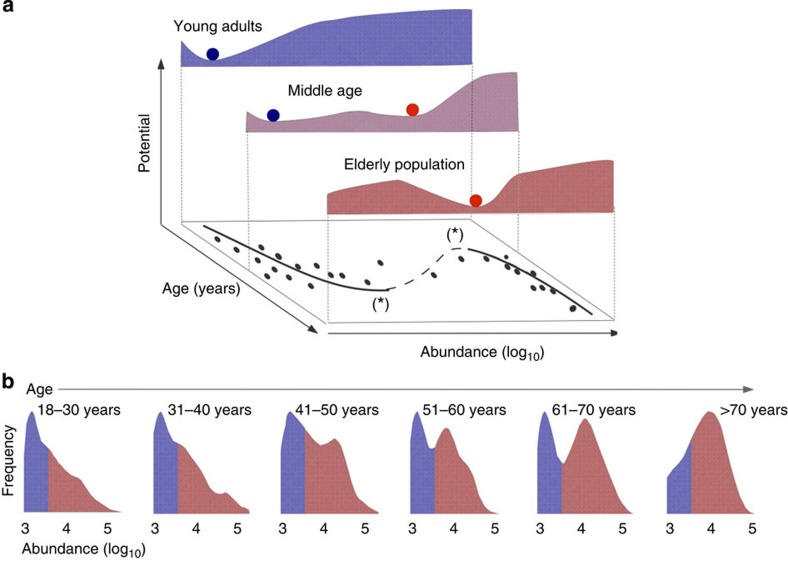
Changing state probabilities associated with ageing. (**a**) A schematic illustration of shifting state probabilities in the UCI group associated with ageing. The UCI abundance data (bottom plane) suggest a catastrophe-fold, where the solid and dashed parts of the curve correspond to stable states and unstable equilibria, respectively. The depth and width of the potential minima indicate decreasing resilience of the system towards the bifurcation points (*) during ageing. (**b**) Observation density in various age groups highlights the associations between the UCI state probabilities and age (sample sizes: 220 (18–30 years); 147 (31–40 years); 192 (41–50 years); 258 (51–60 years); 114 (61–70 years); and 19 (71–77 years)). The alternative states of low (blue) and high (red) abundances are here more clearly pronounced than in the population-level histogram pooled over all age groups (Fig. 1).

The emerging picture is that rather than overarching alternative states corresponding to distinct community types, the natural variation of the human intestinal microbiota is reflected in specific bacterial groups representing ‘tipping elements’ that are only loosely coupled, specific components of the intestinal microbiota that exhibit alternative stable states linked to the overall microbiota composition and host factors. We have borrowed this analogy from the climate system that is now also believed to have distinct tipping elements^11^. An important corollary is that the resilience (the capacity to recover from perturbations) of the alternative states will be influenced by a range of factors that may primarily affect specific subcommunities within the intestinal microbiota, rather than the overall ecosystem. Gradual changes in external or internal factors associated with the ecosystem changes may drive the implicated bistable bacteria towards a tipping point where an abrupt shift between the contrasting alternative states may follow^8^,^30^. Our analysis indicates that such shifts are often associated with changes in a wider set of covarying taxa (Fig. 7). One way to infer how resilience of the microbiota might depend on such factors is to use probability densities of bacterial abundance to estimate the basins of attraction. On the basis of this, we can observe, for instance, that a basin of attraction around an apparently resilient high-abundance state of UCI arises in late middle age (Fig. 8; Supplementary Table 3). This statistically significant association between UCI and age (*P*<0.05; *χ*^2^ test) suggests that the resilience of the low-abundance state decreases with increasing age, and eventually, even a small perturbation may induce a shift to the high abundance state. Moreover, the analysis shows that population-level variation may mask alternative states that are more pronounced in specific subpopulations, such as particular age groups (Fig. 8b). According to a logistic regression model, each age decade increased the odds of the UCI high-abundance state by 2.6±1.2% (95% confidence intervals), but the changes were particularly emphasized in the young and elderly populations. These findings are in line with previous studies that have reported notable changes in the overall microbiota composition in the elderly population^27^,^31^,^32^, and with a recent study where differences at the community level were observed between young and elderly adults, but less during the middle age^31^.

Overall, we observed a range of statistically significant associations between the bimodal taxa and host factors such as ageing, obesity and health status based on logistic regression (*P*<0.05; *χ*^2^ test; see Methods). Among the 1,006 western adults, body mass index (BMI) was negatively associated with UCI and UCII. Age was positively associated with the UCI and UCII, and negatively with the *Dialister* spp. and *Prevotella* groups. Males had higher levels of bacteria belonging to the *Prevotella* group and lower levels of the *B. fragilis* group compared with women. Regarding nationality, the *Prevotella* group was underrepresented in Nordic and Southern European countries and UCII in South Europe, while the *Dialister* spp. and *B. fragilis* groups were particularly common in Northern Europe (Scandinavia, UK and Ireland). Analysis of an additional phylogenetic microarray data set derived from subjects with irritable bowel syndrome and severe obesity indicated association between these health complications and the low-abundance UCI and UCII (see Methods). Metabolic syndrome (MetS) was positively associated with *B. fragilis* (false discovery rate (FDR)<5%), and showed a trend (FDR<20%) towards positive association with *Dialister* spp. and a negative association with the *Prevotella* group (Supplementary Table 4). We did not observe associations between the bimodal groups and type II diabetes, ulcerative colitis or cardiovascular disease in this data collection. Overall, these results suggest that differences in health status can be reflected in changing state probabilities of specific taxa.

In conclusion, these observations provide substantial evidence for bistability in specific taxonomic groups within the human intestinal microbiota, including *Dialister* spp., the *Prevotella* group and the uncultured Clostridiales groups UCI and UCII. These groups exhibited robust bimodality in their abundance distributions, and decreased temporal stability at the intermediate abundance range. A bimodal abundance distribution associated with the *B. fragilis* group was also observed, but we could not confirm the bistability of this group in subsequent analyses. Our findings were supported by independent subsets of the data using different DNA extraction methods and geographical distributions, and in additional data sets from dietary intervention trials, including 46 placebo-treated control subjects. Whereas the observed states were robust to short-term dietary differences, which could not explain the observed population-level bimodal patterns, this does not exclude the possibility that targeted dietary interventions could be potentially used to manipulate specific bimodal groups in a personalized manner; we did observe state shifts in individual subjects following dietary interventions, but these changes were masked by the overall within-state variation in the other subjects and did not reach statistical significance in our limited intervention data. The observed patterns were most pronounced in individual phylotypes and in specific subpopulations, such as different age groups, and linked to the overall ecosystem composition and host health.

## Discussion

Our results demonstrate that despite the dominating nature of gradual variation in bacterial abundances in the intestinal ecosystem, specific bacterial groups form a limited number of contrasting, stable configurations that associate with host physiology and health. Bistability in bacterial abundances can be induced by many factors, such as disruptive selection^33^, competition and cooperative feedback loops^34^, or environmentally or genetically determined host factors. Hence, we believe that the here identified bistable taxa, together with other microbial co-occurrence relationships^26^,^35^,^36^, will be instrumental in providing insight on the variation, regulation and health implications of the intestinal microbiota. Targeting specific subpopulations, as opposed to the daunting complexity and variability of the entire ecosystem, can simplify the characterization and possible manipulation of the intestinal microbiota. The implicated taxa may allow stratification of the subjects based on alternative states that in parallel to gene richness^29^ represent promising novel targets for microbiome-based diagnostics and therapies^37^. Moreover, resetting these ‘bacterial dual in-line package (DIP) switches’ may be a radically new way to approach the rapidly growing number of health issues related to the intestinal microbiota, in analogy to the binary DIP switches from early computer era. Further intervention studies will be needed to address the causal relations with the overall community composition and host factors, and to investigate whether these alternative stable states translate into differential disease susceptibility or drug response of the host.

## Methods

### Sample collection

Phylogenetic microarray data from over 5,000 intestinal samples were available in the in-house MySQL database of the HITChip, a phylogenetic microarray^12^. To investigate the microbiota variability in western adult population, we selected from the HITChip database 1,006 western adults (18–77 years; average BMI 26.7 kg m^−2^; s.d. 5.8 kg m^−2^). To control the potentially confounding effect of other environmental or host factors, we curated the available sample annotations and excluded subjects who had reported diseases or antibiotic treatments, or were living outside western countries (Europe/the United States), and used bootstrap analysis to verify that the results were robust to random sampling fluctuations as described below. A single faecal sample per subject was selected; the first time point was used if multiple samples were available. The subjects cover 15 western countries (Europe and the United States) that were further aggregated into Central European (Belgium, Denmark, Germany, the Netherlands), Eastern European (Poland), Nordic (Finland, Norway, Sweden), South European (France, Italy, Serbia, Spain), UK/Ireland (UK, Ireland) and the United States (the United States) groups. To our knowledge, this is the largest collection of phylogenetic microarray profiling data of the human intestinal microbiota. Due to the retrospective nature of this study, we did not select a particular sample size based on power calculations or related approaches or replicate the experiments but instead included all available data, and performed various statistical tests and verifications in independent subsets of the data to assess the significance and robustness of the findings.

### Microbiota profiling

Culture-independent techniques for characterizing intestinal microbiota include phylogenetic microarrays and sequencing-based approaches^38^. Phylogenetic microarrays can be used to quantify with high accuracy the known (previously detected) bacteria including the low-abundance phylotypes that often remain undetectable with conventional sequencing depths^39^. Our analysis is based on the phylogenetic HITChip microarray^12^, produced by Agilent Technologies (Santa Clara, CA, USA), that provides a sensitive analysis platform to assess differences in relative abundance of intestinal bacteria. The HITChip microarray targets the V1 and V6 hypervariable regions of 16S rRNA gene, and can detect 1,033 species-like bacterial phylotypes (>98% sequence similarity in the 16S rRNA gene) that represent the majority of the bacterial diversity of the human intestine^12^. For the analyses, hybridization signals were summarized to 130 genus-like phylogenetic groups (>90% sequence similarity in the 16S rRNA gene) that are referred to as species and relatives, the latter being shortened as ‘*et rel.*’^12^. The HITChip microarray allows highly reproducible (98±2% Pearson correlation across technical replicates) and deep analysis (reproducible detection of phylotype abundances below a 0.1% relative abundance level) of the phylotype composition of intestinal samples, comparable to 200,000 next-generation sequencing reads per sample^39^. Hence, the standardized analytical pipeline for the HITChip microarray data collection provides advantage over sequencing-based approaches, where the comparable collections of intestinal microbiota profiling data are smaller, and integration of data from individual studies is more challenging, for example, due to the use of different primers, continuously changing sequencing platforms and protocols. The faecal samples, collected in the context of clinical trials, were typically taken at home, frozen at −20 °C, delivered to the study centre within 24 h and stored at −80 °C. The DNA was extracted and prepared by amplification of the full-length 16S rRNA gene, followed by transcription and labelling of the resultant RNA with Cy3 and Cy5 before fragmentation and hybridization on the array^12^. The signal intensity data from the microarray hybridizations were collected using the G2505C scanner by Agilent and preprocessed using an in-house MySQL database and custom R scripts. Each scanner channel from the array was spatially normalized using polynomial regression, followed by outlier detection and filtering in each set of probes with a *χ*^2^ test. The intensities were sample-wise quantile normalized and averaged to a single value for each probe–sample pair. Each sample was hybridized on at least two replicates to ensure reproducibility. Between-sample normalization was performed at probe level with the min–max normalization using 0.5% quantiles estimated from the data. Each targeted genus-like group is probed by multiple (median 19) oligos. Logarithmized (log_10_) signal of the probes targeting the same phylotype were summarized with Robust Probabilistic Averaging^40^,^41^ (fRPA; RPA R package). The log_10_-transformed signals were used as a proxy for bacterial logarithmic abundance. As a proxy for relative abundance, we used the fraction of the total probe signal calculated for the probes targeting a given taxonomic group.

### Bacterial abundance types

We categorized the 130 genus-like groups into characteristic ‘abundance types’ to gain an overview of their overall population-level variability. The abundance types included rare taxa and prevalent taxa with symmetric, (left- and right-) skewed and bimodal distributions (Fig. 2; Supplementary Table 1). The bimodal taxa were identified as described below, including verification in multiple data sets. For the other genus-like groups, the categorization was based on the 401 unique subjects whose faecal samples were processed with the repeated bead beating protocol that is based on mechanical cell disruption method for DNA extraction, and that we have found to be superior to alternative methods in terms of DNA extraction efficiency^25^. Altogether, 60 bacterial groups were abundant and prevalent, exceeding HITChip log_10_ signal of 3.0 in >20% of the subjects; the remaining 70 groups were considered rare. The 34 prevalent groups with symmetric log abundance (absolute skewness <0.5; e1071 CRAN R package) exhibited relatively constant logarithmic abundances across subjects, fluctuating symmetrically around a mean value. The skewed types included both rare and prevalent bacteria with both left- and right-skewed types (skewness <−0.5 and >0.5, respectively).

### Potential analysis

We used potential analysis as the primary state identification method. This approach is based on the theory of stochastic dynamical systems, and was recently applied to identify alternative stable states in climatology and forest ecosystems^13^,^14^. In the following, we provide a brief summary of the underlying theory and assumptions.

Let us assume an underlying stochastic system that has a potential function of the form


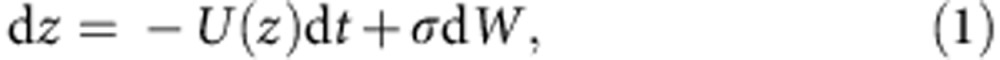


where *U*(*z*) is the potential function, *z* is the state variable (in our case, the microbe log_10_ abundance), *σ* is the noise level and d*W* is a noise term (Wiener process). The minima of the potential function correspond to stable states of the system. The corresponding Fokker–Planck equation connects the potential of this model to the probability density of the state variable *z*. The potential *U* is given by:


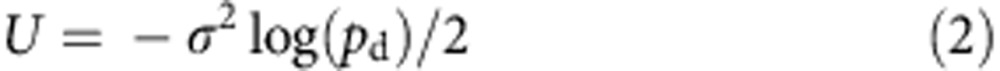


where *p*_d_ is the empirically estimated probability density function of the state variable *z*. An example of the estimated probability density for the bimodal *Dialister* spp. in our data is shown in Supplementary Fig. 3. As our primary interest is in qualitative analysis of the potential function shape, we scaled the potential to the noise level (*U*/*σ*^2^) (ref. 13). We estimated the probability density with Gaussian kernels (the R density function) and automated kernel width adjustment with Scott's method^42^ (the *bw* function in the R stats package), giving the bandwidth *h*=1.06 *s*/*n*^1/5^, where *s* is the s.d. of the data points and *n* is the sample size. We adjusted this bandwidth with a factor of 0.8 to achieve more sensitive detection of multimodality. We estimated the local minima and maxima numerically and reduced spurious findings by requiring that the difference between local minima and the closest maxima was larger than a threshold value^13^ (0.005 in our analysis) and with a minimum density of 0.1. We used the number of local minima of the potential function to quantify the number of distinct modes^14^.

### Bimodality detection

We implemented the potential analysis as a state detection methodology^13^,^14^ in the earlywarnings R package (http://www.early-warning-signals.org/; v. 1.0.59; livpotential_ews function). To avoid spurious findings associated with random sampling fluctuations and to quantify the robustness of bimodal patterns, we repeated this analysis in 100 bootstrap data sets, and selected for further analysis the taxonomic groups that exhibited consistent evidence for multimodality in ≥90% of the bootstrap data sets (Supplementary Note 5). While the estimated number of modes in some cases varied between the bootstrap data sets, the evidence was the strongest for two alternative states in all multimodal groups. Hence, we used the overall fraction of bootstrap samples that indicated deviation from unimodality to quantify the evidence for bimodality. Our sample collection included samples from multiple studies extracted using different DNA extraction methods, which could affect bimodality detection in a taxon-specific manner^25^. To control this, we confirmed the bimodality of the reported six bimodal groups in the subset of 401 samples extracted using the mechanical lysis-based DNA extraction method (repeated bead beating)^25^. We also assessed bimodality in the subset of 287 samples processed using enzymatic lysis. The UCII group had a moderate (83%) bootstrap support for bimodality, whereas bimodality of the *B. fragilis* group was not supported in this smaller data set (31% bootstrap support). The bimodal patterns in *Dialister* spp., *P. melaninogenica*, *P. oralis* and UCI were verified in 100% of the bootstrap samples also in this data set. For the final analysis, we included the *B. fragilis*, *Dialister* spp., *P. melaninogenica*, *P. oralis*, UCI and UCII groups whose bimodality was detected in the overall data collection and in at least one of the two distinct DNA extraction method subsets with >90% bootstrap support (Supplementary Note 6). An independent analysis of bimodality in these univariate observations of bacterial abundance was performed using the PSI (with *M*=20 random splits into training and test sets; fpc R package) based on the partition around medoids clustering and Euclidean distance^10^,^15^.

### Tipping point

The tipping point is here defined as the particular bacterial abundance value that maximizes the potential function of a bistable system; this corresponds to an unstable threshold between the contrasting alternative stable states, which correspond to the local minima of the potential function. The potential minima and maxima were determined by potential analysis. We used the average over the different bootstrap data sets as the final tipping point estimate; this provides increased robustness against random sampling fluctuations.

### Temporal analysis

To investigate the temporal stability of the observed alternative states, we analysed 78 subjects from the main cohort with additional follow-up data from dietary intervention trials; 28% of the follow-up samples were subject to dietary intervention but no significant changes following the dietary intervention were seen in the bimodal taxa, except for the UCI and UCII groups (*P*<0.05; *t*-test). The median age of the subjects was 49 years (27–63 years); the median BMI 33 kg m^−2^ (18–43 kg m^−2^); most subjects (*n*=65; 86%) were females from Finland (*n*=43; 55%) and Belgium (*n*=32; 41%); and 81% of the subjects had 2–3 time points; the remaining 19% had 4–5 time points. Most (95%) time points were within 1–9 months from the baseline (mean 3.5 months), with the average follow-up interval (time between consecutive time points) of 2.4 months. Despite some variability between subjects, the follow-up times were within a similar time span and the differences in the follow-up times did not explain differences in the observed fluctuations between time points, as these two variables were not significantly correlated in our data (|*ρ*|<0.05; *P*>0.05; upper tail (AS89) test). We quantified the stability at the intermediate abundance range by calculating the correlation between the distance from the intermediate (50%) abundance quantile and the observed shift between the consecutive time points; negative correlations indicate instable regions where larger shifts in microbial abundance are observed on average. In addition, we approximated the state shift frequencies in subjects with varying follow-up times based on Kaplan–Meier survival analysis (the survival R package), which gives an estimate of the fraction of subjects remaining in their initial state as a function of time based on limited observations with varying follow-up times. We used the estimated fraction of subjects remaining in their original state at the 3-month time point to quantify state stability (Supplementary Note 7). Since the observed state shifts could arise from natural, continuous fluctuations in bacterial abundance rather than abrupt shifts between alternative stable states, we used the same procedure to estimate how often state shifts would be observed in the other prevalent taxa based on simulated states of low and high abundance based on the same abundance quantiles to determine the ‘tipping point’ between the two states, and compared the bistable groups with the other prevalent taxa (Supplementary Fig. 2b).

### Community-level analysis

We assessed the evidence for alternative states at the overall ecosystem level based on PCA (Supplementary Fig. 7a) and the partition around medoids multivariate clustering with Jensen–Shannon dissimilarity, coupled with the PSI to determine the optimal cluster number as this combination was earlier shown to have a good overall performance^10^ (Supplementary Note 8). In addition, we used principal coordinates analysis (that is, classical multidimensional scaling; cmdscale function of the R stats package)^43^ as a complementary nonlinear approach for community-level visualization^10^ based on generalized UniFrac distances^28^ (GUniFrac function of the R GUniFrac package^44^) that take into account the phylogenetic structure of the community, quantified by HITChip probe-level analysis (Supplementary Fig. 7b).

### Short-term dietary interventions

Whereas dietary information was not available for our main data set, we analysed the effects of short-term dietary interventions in an additional data set from the HITChip database consisting of 138 subjects sampled during dietary intervention studies, including three previously published^16^,^17^,^45^ and two unpublished dietary intervention trials: (i) 52 Finnish adults fulfilling the criteria for MetS that were subjected to qualitative changes in carbohydrate intake^16^ (50% of the samples in a control group); (ii) 33 obese Belgian women from a prebiotic (inulin-type fructan) intervention study^45^ (50% of the samples in a control group); (iii) 31 obese Finnish subjects with weight-loss diet (HITChip project ‘ISRCTN67529475’); (iv) 8 Norwegian subjects with a vegan diet (HITChip project ‘Vegan’); and (v) 14 obese British males with fibre-supplemented and weight-loss diets^17^. In addition to the dietary intervention subjects, this data collection included 46 placebo-treated control subjects.

### Associations with host parameters

We quantified associations between the bimodal taxa and host factors by using subject metadata (age, BMI, sex, nationality) and DNA extraction method as fixed effects in multiple logistic regression to predict the state of the corresponding bacteria in each subject. The significance of each parameter was estimated by log-ratio (*χ*^2^) test comparing the models with and without the corresponding factor in predicting the state (the GLM function in the R stats package). Missing annotations were omitted, and the FDR was estimated to correct for multiple testing based on the Benjamini–Hochberg method with the p.adjust function (R stats package).

### Health associations

To investigate the associations between the alternative states and health status, we compared the 1,006 adults with no reported health complications with additional samples in the HITChip database representing western adults with irritable bowel syndrome (*n*=106), MetS (*n*=66), type II diabetes (*n*=78), ulcerative colitis (*n*=62) and cardiovascular disease (*n*=45). In addition, we divided the non-compromised subjects into severe obese (BMI≥35; *n*=136) and other (BMI<35; *n*=870) subjects. We used multiple logistic regression to predict disease occurrence from logarithmic abundance of each bimodal bacterial group. The DNA extraction method, age, BMI and sex were included in the model as potentially confounding variables. The *P* values from log-ratio test were corrected using the Benjamini–Hochberg procedure. For exploratory purposes, associations with FDR<20% were considered to establish a trend and FDR<5% to be significant (Supplementary Table 4). We compared the performance of the logistic regression model with and without bacterial abundance based on receiver operator characteristic analysis to detect disease occurrence; the average area under curve (AUC) values were derived from fivefold cross-validation. While our analysis suggests significant association between MetS and specific taxa, the differences in the AUC values between the models with and without bacterial abundance were small (0.1 increase with bacterial abundance) in the MetS group, indicating limited predictive ability in our data to distinguish the effects of MetS from the effects of age, sex and BMI. In severe obesity, inclusion of bacterial abundance notably improved disease prediction (AUC 0.58 versus 0.69 for UCI; 0.60 versus 0.68 for UCII).

## Author contributions

L.L., M.S. and W.M.d.V. designed the study. L.L. carried out data processing and statistical analysis, coordinated the study and prepared the manuscript. A.S., L.L., J.S. and W.M.d.V. contributed to acquisition of data. J.S. contributed to data analysis. All authors contributed to interpreting the results and writing the manuscript.

## Additional information

**Accession codes**: The phylogenetic HITChip microarray data matrix and anonymized subject metadata have been deposited in the Dryad Digital Repository: http://doi.org/10.5061/dryad.pk75d

**How to cite this article:** Lahti, L. *et al*. Tipping elements in the human intestinal ecosystem. *Nat. Commun.* 5:4344 doi: 10.1038/ncomms5344 (2014).

## Supplementary Material

Supplementary Information Supplementary Figures 1-7, Supplementary Tables 1-4, Supplementary Notes 1-8 and Supplementary References
