# Supplementary material for: Tipping elements in the human intestinal ecosystem
Source: Nat Commun. 2014 Jul 8;5:4344. doi: 10.1038/ncomms5344 (PMC4102116; doi:10.1038/ncomms5344)
Supplement: Supplementary Information — Supplementary Figures 1-7, Supplementary Tables 1-4, Supplementary Notes 1-8 and Supplementary References [file ncomms5344-s1.pdf]

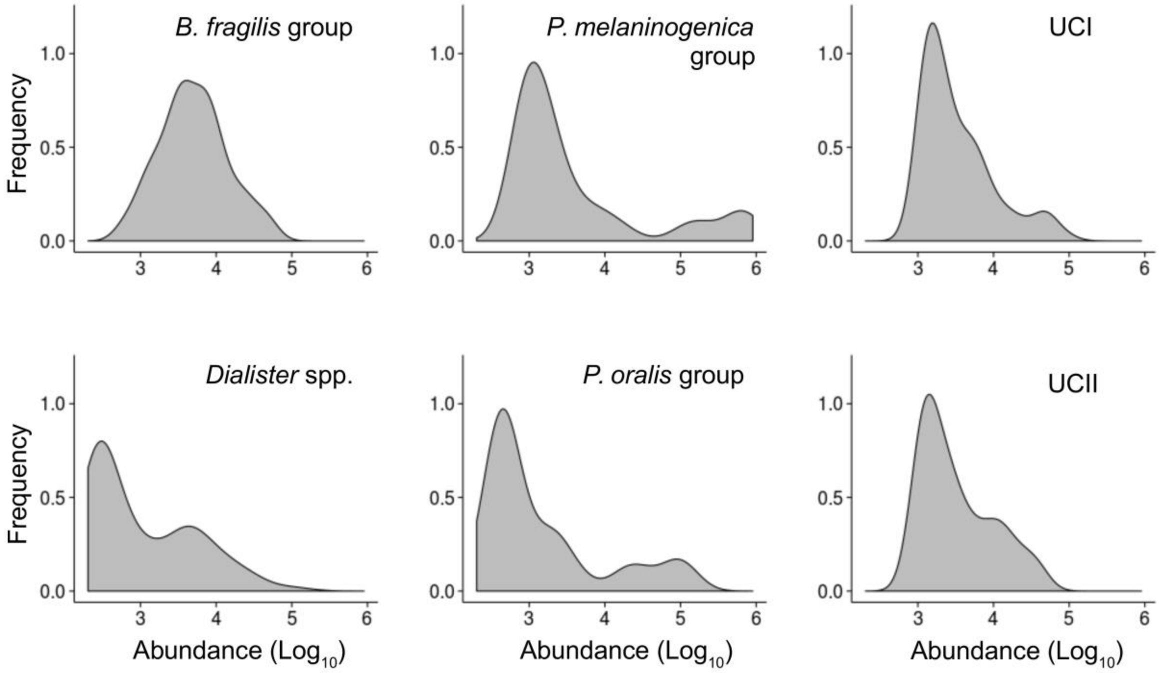

Supplementary Fig. 1 Abundance distributions for control subjects Logarithmic abundance distributions across 46 control subjects (*n*=92 samples) from dietary intervention trials (Methods) show indications of bimodality. These control subjects received placebo, and were controlled for other external factors including probiotics and antibiotics use. The *B. fragilis* group is an exception with no indications of bimodality.

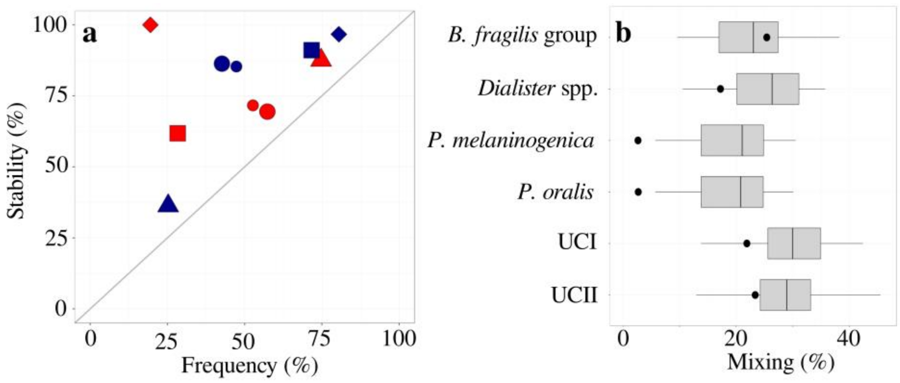

Supplementary Fig. 2 State frequency and stability (a) State frequency (fraction of subjects associated with the state) among the 1,006 western adults (horizontal axis) correlates with short-term stability of the state in the 78 follow-up subjects (vertical axis). The stability is quantified by the fraction of subjects remaining in their original state over a three-month interval, as estimated by Kaplan-Meier survival analysis (Methods). All bi-stable groups appear more stable than expected by random state allocation between the consecutive time points (gray line), the *Prevotella* group (♦) being the most stable (>97%). Symbols: *B. fragilis* group (▲), *Dialister* spp. (■), *Prevotella* group (♦), UCI (•), UCII (●); the blue and red color indicate the low- and high-abundance states, respectively. **(b)** State mixing within each bi-stable group compared to the expected mixing; the mixing is defined as the overall fraction of subjects who exhibit a state switch during the study interval. Since the observed state mixing could reflect natural, continuous fluctuations in bacterial abundance rather than abrupt shifts between contrasting stable states, we compared the mixing rates in the bi-stable taxa (black dots) to the 54 other prevalent, unimodal taxa (boxplots) with simulated low- and high-abundance states constructed based on the same population frequencies than in the corresponding bi-stable group. The *P. oralis* and *P. melaninogenica* groups appear the most stable, with less mixing than in any other group (100%). Also *Dialister* spp. (83%), UCI (93%) and UCII (78%) exhibit less mixing than most unimodal groups based on analogous, simulated tipping points. The *B. fragilis* group (35%) is an exception that appears less stable than most other taxa.

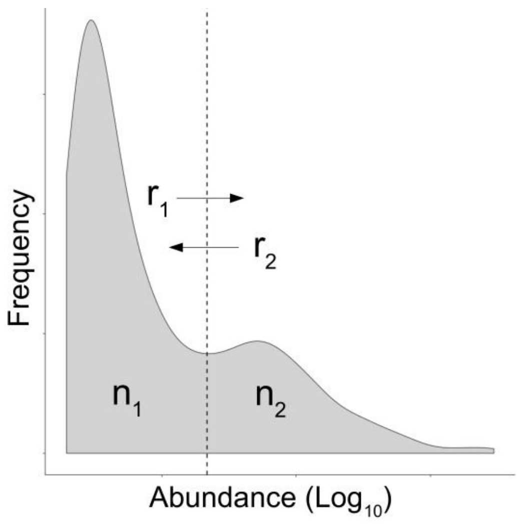

Supplementary Fig. 3 Temporal dynamics of a bi-stable abundance distribution The logarithmic abundance distribution for *Dialister* spp. exhibits two peaks of low and high abundance with the estimated state frequencies of n_1=72% and n_2=28% across the 1,006 western adults, respectively. In stationary state, the flow between the two states is balanced, and the ratio of the switching rates *r* should be inversely related to state frequencies *n* ($r_1/r_2 = n_2/n_1$) assuming a stationary continuous-time Markov process. Hence the more frequent states are relatively more stable in the stationary state, while the absolute switching rates determine the overall mixing between the states during a given time interval. The tipping point (dashed line) marks the intermediate region between the alternative states of low and high abundance. In bi-stable systems the observations around this region have reduced stability[1].

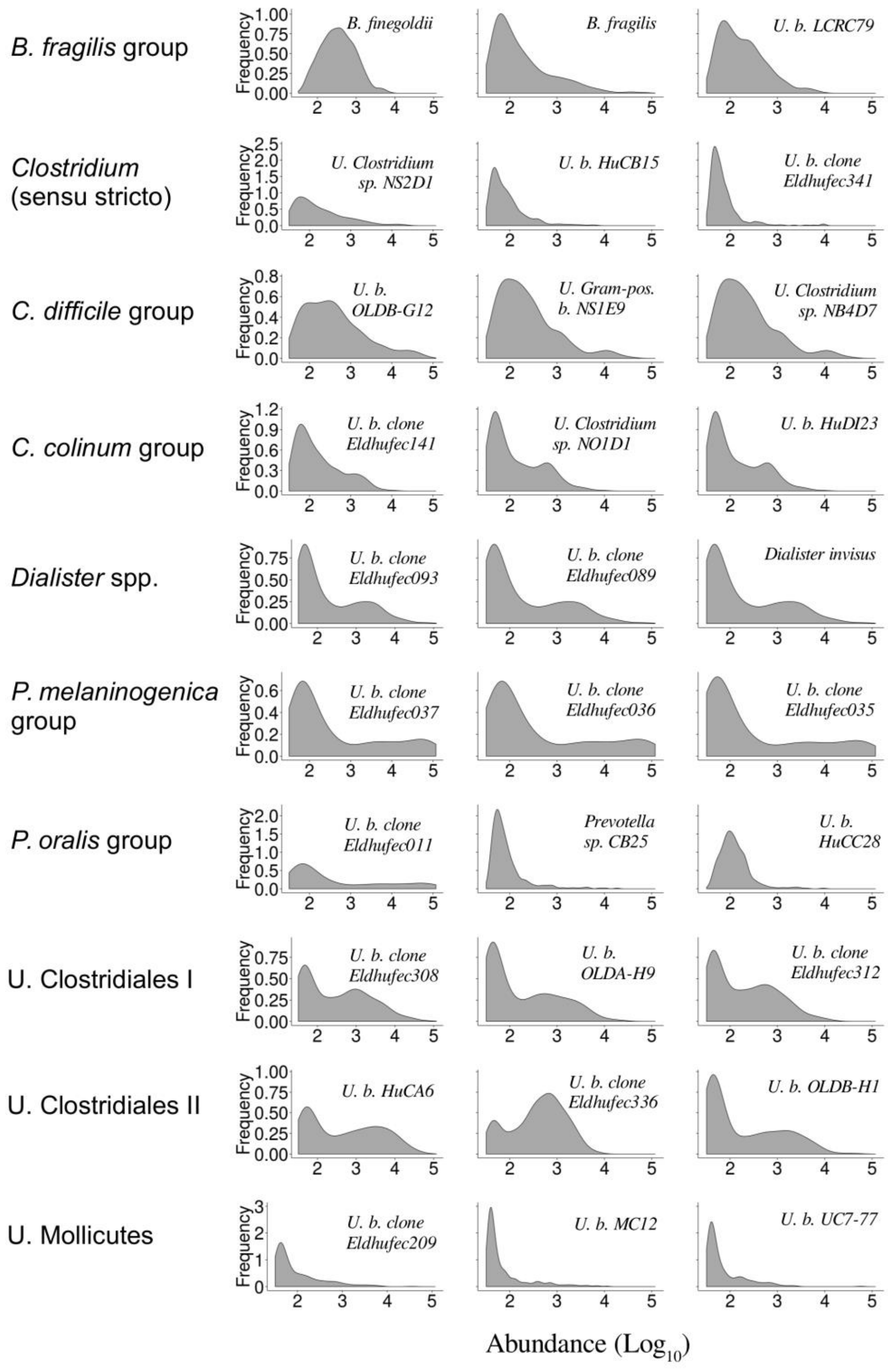

Supplementary Fig. 4 Phylotype abundances for selected genus-like groups Logarithmic abundance distributions for the three most prevalent phylotypes within the genus-like groups that show indications for bi-stability (main Fig. 4). Prevalent phylotypes based on our criteria (see Methods) are identified within the *Dialister* spp., relatives of *P. oralis and P. melaninogenica,* UCI and UCII groups. Abbreviations: Uncultured ('U.'); bacteria ('b.').

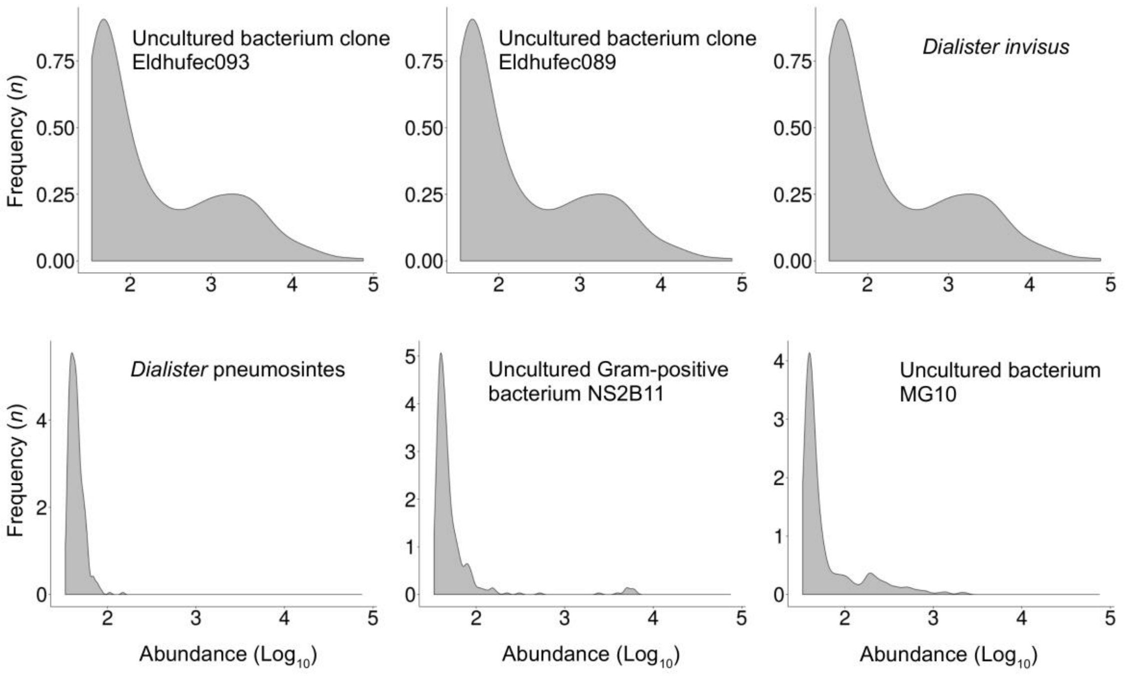

Supplementary Fig. 5 Abundance distributions for the phylotypes within *Dialister* spp.

Strong bimodality in the abundance distributions is observed in three phylotypes within the *Dialister* spp. (Uncultured bacterium clones Eldhufec093 and Eldhufec089; and *D. invisus*).

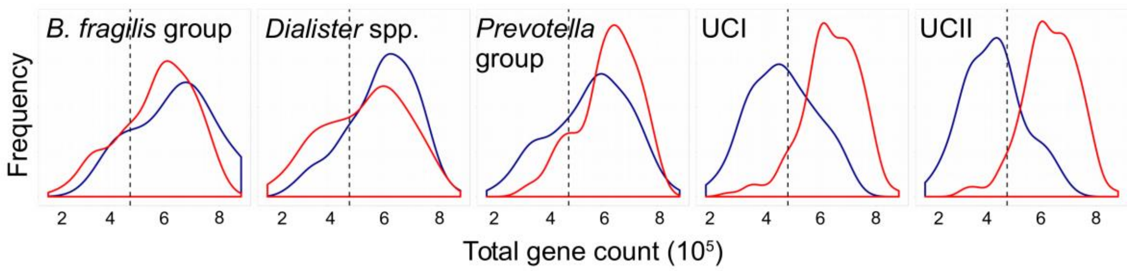

Supplementary Fig. 6 Alternative states and gene richness Total gene count for 255 subjects (39-72 years) in a metagenomic sequencing study[2], where a bimodal gene count was reported; the corresponding threshold between the low and high gene count is indicated by the vertical dashed line. The total gene count distributions are illustrated separately for the subjects associated with the low- (blue) and high- (red) abundance state of each bi-stable group. The metagenomic gene count is significantly associated with all bi-stable groups (P<0.01; Wilcoxon test) except the *B. fragilis* group (P=0.2). *Dialister* spp. is associated with the low gene count[2]; the *Prevotella*, UCI and UCII groups are associated with high gene count.

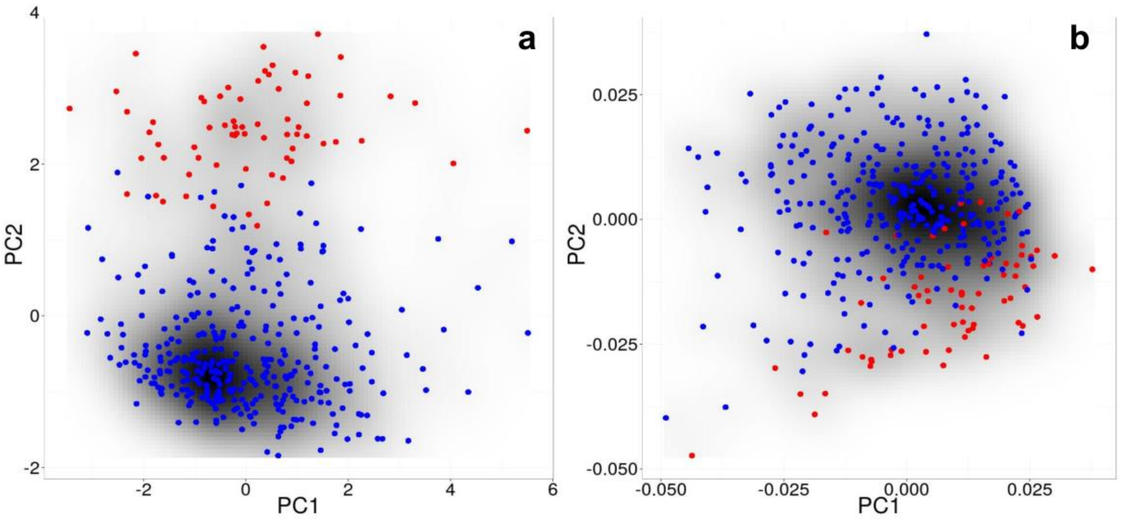

Supplementary Fig. 7 Community-level variation (a) Visualization of the two main axes of Principal Component Analysis based on the 130 genus-like groups across the 401 samples extracted with the mechanical lysis. The gray shading indicates the density of the data points. The second principal axis, driven by the *Prevotella* group (relatives of *P. melaninogenica* and *P. oralis*), explains 13% of the overall variance in the data. **(b)** Visualization of the same data based on Principal Coordinates Analysis with un-weighted UniFrac distances. The blue and red colors indicate subjects with the low and high abundance state of the *Prevotella* group based on our analysis, respectively.

Symmetric (34)	Akkermansia; *Allistipes et rel.*; *Anaerostipes caccae et rel.*; *Anaerovorax odorimutans et rel.*; *Bacteroides ovatus et rel.*; *Bacteroides plebeius et rel.*; *Bacteroides uniformis et rel.*; *Bacteroides vulgatus et rel.*; Bifidobacterium; *Bryantella formatexigens et rel.*; *Clostridium cellulosi et rel.*; *Clostridium leptum et rel.*; *Clostridium nexile et rel.*; *Clostridium orbiscindens et rel.*; *Clostridium sphenoides et rel.*; *Clostridium stercorarium et rel.*; *Clostridium symbiosum et rel.*; *Coprococcus eutactus et rel.*; *Dorea formicigenerans et rel.*; *Eubacterium hallii et rel.*; *Eubacterium rectale et rel.*; *Eubacterium ventriosum et rel.*; *Lachnobacillus bovis et rel.*; *Oscillospira guillermondii et rel.*; Outgrouping clostridium cluster XIVa; *Oxalobacter formigenes et rel.*; *Papillibacter cinnamivorans et rel.*; *Roseburia intestinalis et rel.*; *Ruminococcus bromii et rel.*; *Ruminococcus callidus et rel.*; *Ruminococcus gnavus et rel.*; *Ruminococcus obeum et rel.*; *Sporobacter termitidis et rel.*; *Tannerella et rel.*
Left-skewed (4)	*Butyrivibrio crossotus et rel.*; *Faecalibacterium prausnitzii et rel.*; *Lachnospira pectinoschiza et rel.*; *Subdoligranulum variable et rel.*
Right-skewed (16)	*Anaerotruncus colihominis et rel.*; *Bacteroides splachnicus et rel.*; *Bacteroides stercoris et rel.*; *Clostridium (sensu stricto)*; *Clostridium colinum et rel.; Clostridium difficile et rel.*; Collinsella; *Eubacterium biforme et rel.*; *Lactobacillus plantarum et rel.*; *Parabacteroides distasonis et rel.*; *Prevotella tannerae et rel.*; *Ruminococcus lactaris et rel.*; *Streptococcus bovis et rel.*; *Streptococcus mitis et rel.*; *Sutterella wadsworthia et rel.*; Uncultured Mollicutes
Bimodal (6)	*Bacteroides fragilis et rel.*; *Dialister* spp.; *Prevotella melaninogenica et rel.*; *Prevotella oralis et rel.*; Uncultured Clostridiales I; Uncultured Clostridiales II
Rare (70)	Actinomycetaceae; Aerococcus; Aeromonas; *Alcaligenes faecalis et rel.*; Anaerobiospirillum; Anaerofustis; Aneurinibacillus; Aquabacterium; *Asteroleplasma et rel.*; Atopobium; Bacillus; *Bacteroides intestinalis et rel.*; *Bilophila et rel.*; Brachyspira; *Bulleidia moorei et rel.*; Burkholderia; Campylobacter; *Catenibacterium mitsuokai et rel.*; *Clostridium felsineum et rel.*; *Clostridium ramosum et rel.*; *Clostridium thermocellum et rel.*; *Coprobacillus catenaformis et rel.*; Corynebacterium; *Desulfovibrio et rel.*; *Eggerthella lenta et rel.*; *Enterobacter aerogenes et rel.*; Enterococcus; *Escherichia coli et rel.*; *Eubacterium cylindroides et rel.*; *Eubacterium limosum et rel.*; *Eubacterium siraeum et rel.*; Fusobacteria; Gemella; Granulicatella; Haemophilus; Helicobacter; *Klebisiella pneumoniae et rel.*; *Lactobacillus catenaformis et rel.*; *Lactobacillus gasseri et rel.*; *Lactobacillus salivarius et rel.*; Lactococcus; Leminorella; *Megamonas hypermegale et rel.*; *Megasphaera elsdenii et rel.*; Methylobacterium; Micrococcaceae; *Mitsuokella multiacida et rel.*; Moraxellaceae; Novosphingobium; Oceanospirillum; *Peptococcus niger et rel.*; *Peptostreptococcus anaerobius et rel.*; *Peptostreptococcus micros et rel.*; *Phascolarctobacterium faecium et rel.*; *Prevotella ruminicola et rel.*; Propionibacterium; *Proteus et rel.*; Pseudomonas; Serratia; Staphylococcus; *Streptococcus intermedius et rel.*; Uncultured Bacteroidetes; Uncultured Chroococcales; Uncultured Selenomonadaceae; Veillonella; Vibrio; *Weissella et rel.*; *Wissella et rel.*; Xanthomonadaceae; *Yersinia et rel.*

Supplementary Table 1 Bacterial abundance types Characteristic abundance types of the 130 genus-like bacterial groups quantified by the phylogenetic HITChip microarray. The genus-like groups (>90% sequence similarity in the 16S rRNA gene) are referred to as *type species* and relatives, the latter being shortened as "*et rel.*"[3]. The symmetric, skewed, bimodal, and rare abundance types are illustrated in main Fig. 2.

	Genus-like group	Species-level phylotypes	Accession number
Bacteroidetes	*Bacteroides fragilis et rel.*	bacterium adhufec23	AF132251
		bacterium adhufec355	AF132263
		Bacteroides thetaiotaomicron	L16489
		Bacteroides fragilis	M11656
		Uncultured bacterium MR34	AY916210
		Uncultured bacterium Z091	AY916178
		Uncultured bacterium LCLC32	AF499834
		Uncultured bacterium LCRC79	AF499852
		Bacteroides finegoldii	AB222699
		Bacteroides nordii	AY608697
		Bacteroides salyersiae	AY608696
	Prevotella melaninogenica et rel.	bacterium adhufec235	AF132249
		bacterium adhufec43	AY920192
		Prevotella albensis	AJ011683
		Prevotella intermedia	AF414821
		Prevotella salivae	AB108826
		Prevotella sp. BI-42	AJ581354
		Prevotella sp. CB7	AB064923
		Prevotella melaninogenica	L16469
		Uncultured bacterium clone Eldhufec008	AY919883
		Uncultured bacterium clone Eldhufec007	AY919882
		Uncultured bacterium clone Eldhufec038	AY919913
		Uncultured bacterium clone Eldhufec037	AY919912
		Uncultured bacterium clone Eldhufec036	AY919911
		Uncultured bacterium clone Eldhufec035	AY919910
		Uncultured bacterium B176	AY916316
		Uncultured bacterium M107	AY916148
		Uncultured bacterium OLDC-A6	AB099766
		Uncultured *Prevotella* sp. NB2E9	AB064832
	Prevotella oralis et rel.	*Prevotella buccalis*	L16476
		Prevotella oralis	L16480
		Prevotella sp. CB25	AB064924
		Uncultured bacterium HuCC28	AJ315483
		Uncultured bacterium clone Eldhufec011	AY919886
		Uncultured bacterium clone Eldhufec043	AY919918
Firmicutes;	*Dialister* spp.	*Dialister invisus*	AY162469
Clostridium cluster IX		*Dialister pneumosintes*	X82500
		Uncultured Gram-positive bacterium NS2B11	AB064859
		Uncultured bacterium clone Eldhufec093	AY919968
		Uncultured bacterium clone Eldhufec089	AY919964
		Uncultured bacterium MG10	AY982155
Firmicutes;	Uncultured Clostridiales I	Uncultured human gut bacterium JW2B4	AB080852
Uncultured Clostridiales		Uncultured human gut bacterium JW2F12	AB080851
		Uncultured bacterium OLDA-C7	AB099786
		Uncultured bacterium OLDA-F6	AB099785
		Uncultured bacterium OLDA-F7	AB099784
		Uncultured bacterium OLDA-H9	AB099782
		Uncultured bacterium OLDB-A9	AB099783
		Uncultured bacterium OLDCA-1	AB099781
		Uncultured bacterium C118	AY916326
		Uncultured bacterium C257	AY916329
		Uncultured bacterium C627	AY916340
		Uncultured bacterium D049	AY916352
		Uncultured bacterium D279	AY916363
		Uncultured bacterium D693	AY916381
		Uncultured bacterium LH65	AY916208
		Uncultured bacterium M220	AY916150
		Uncultured bacterium M233	AY916151
		Uncultured bacterium M412	AY916156
		Uncultured bacterium M621	AY916165

	Uncultured bacterium MF22	AY916236
	Uncultured bacterium MF35	AY916239
	Uncultured bacterium MG86	AY916291
	Uncultured bacterium NH06	AY916173
	Uncultured bacterium clone Eldhufec308	AY920183
	Uncultured bacterium clone Eldhufec312	AY920187
	Uncultured bacterium clone Eldhufec314	AY920189
	Uncultured bacterium clone Eldhufec316	AY920191
	Uncultured bacterium clone Eldhufec317	AY920192
	Uncultured bacterium UC7-9	AJ608227
	Uncultured bacterium UC7-127	AJ608249
Uncultured Clostridiales II	Uncultured human gut bacterium JW2H12	AB080880
	Uncultured bacterium OLDB-C2	AB099778
	Uncultured bacterium C736	AY916346
	Uncultured bacterium LQ86	AY916269
	Uncultured bacterium M501	AY916160
	Uncultured bacterium clone Eldhufec333	AY920208
	Uncultured bacterium clone Eldhufec332	AY920207
	Uncultured bacterium cadhufec18c08	AF530351
	Uncultured bacterium cadhufec17f05	AF530343
	Uncultured bacterium OLDB-H1	AB099779
	Uncultured bacterium OLDB-F4	AB099777
	Uncultured bacterium OLDC-A2	AB099780
	Uncultured bacterium C583	AY916338
	Uncultured bacterium C655	AY916341
	Uncultured bacterium D191	AY916361
	Uncultured bacterium K342	AY916195
	Uncultured bacterium M403	AY916155
	Uncultured bacterium MH87	AY916298
	Uncultured bacterium MM92	AY916304
	Uncultured bacterium HuCA6	AJ408962
	Uncultured bacterium clone Eldhufec328	AY920203
	Uncultured bacterium clone Eldhufec329	AY920204
	Uncultured bacterium clone Eldhufec334	AY920209
	Uncultured bacterium clone Eldhufec336	AY920211
	Uncultured human gut bacterium JW1H11	AB080881
	Uncultured human gut bacterium JW1B2	AB080879

Supplementary Table 2 Phylotype-level characterization of the bimodal groups

Cultivated species and uncultured phylotypes (≥98% 16S rRNA gene sequence similarity) that constitute the six bimodal genus-like phylogenetic groups targeted by the HITChip microarray[3]. The right-most column lists the corresponding NCBI accession numbers.

UCI	RDP sequence	HITChip phylotype name	Accession
	Firmicutes; Clostridia; Clostridiales; *Ruminococcaceae*; *Acetivibrio*		
	uncultured bacterium; C118	uncultured bacterium C118	AY916326
	uncultured bacterium; Eldhufec308	Uncultured bacterium clone Eldhufec308	AY920183
	uncultured bacterium; Eldhufec312	Uncultured bacterium clone Eldhufec312	AY920187
	uncultured bacterium; Eldhufec314	Uncultured bacterium clone Eldhufec314	AY920189
	uncultured bacterium; Eldhufec317	Uncultured bacterium clone Eldhufec317	AY920192
	uncultured bacterium; D049	uncultured bacterium D049	AY916352
	Uncultured bacterium; M220	uncultured bacterium M220	AY916150
	uncultured bacterium; MF22	uncultured bacterium MF22	AY916236
	uncultured bacterium; MF35	uncultured bacterium MF35	AY916239
	uncultured bacterium; NH06	uncultured bacterium NH06	AY916173
	Firmicutes; Clostridia; Clostridiales; *Ruminococcaceae*; unclassified *Ruminococcaceae*		
	uncultured bacterium; Eldhufec316	Uncultured bacterium clone Eldhufec316	AY920191
	uncultured bacterium; C257	uncultured bacterium C257	AY916329
	uncultured bacterium; C627	uncultured bacterium C627	AY916340
	uncultured bacterium; M233	uncultured bacterium M233	AY916151
	uncultured bacterium; M412	uncultured bacterium M412	AY916156
	uncultured bacterium; MG86	uncultured bacterium MG86	AY916291
	uncultured bacterium; OLDA-F6	uncultured bacterium OLDA-F6	AB099785
	uncultured bacterium; UC7-127	Uncultured bacterium UC7-127	AJ608249
	uncultured bacterium; UC7-9	Uncultured bacterium UC7-9	AJ608227
	Firmicutes; Clostridia; Clostridiales; unclassified Clostridiales		
	uncultured bacterium; D279	uncultured bacterium D279	AY916363
	uncultured bacterium; D693	uncultured bacterium D693	AY916381
	uncultured bacterium; LH65	uncultured bacterium LH65	AY916208
	uncultured bacterium DGGE gel band; 128-BL-00-B1	uncultured bacterium M621	AY574526
	uncultured bacterium; OLDA-C7	uncultured bacterium OLDA-C7	AB099786
	uncultured human intestinal bacterium; JW2B4	uncultured human gut bacterium JW2B4	AB080852
	uncultured human intestinal bacterium; JW2F12	uncultured human gut bacterium JW2F12	AB080851
	Firmicutes; unclassified Firmicutes		
	uncultured bacterium; OLDB-A9	uncultured bacterium OLDB-A9	AB099783
	uncultured bacterium; OLDA-H9	uncultured bacterium OLDA-H9	AB099782
	unclassified Bacteria		
	uncultured bacterium; OLDC-A1	uncultured bacterium OLDCA-1	AB099781
	uncultured bacterium; OLDA-F7	uncultured bacterium OLDA-F7	AB099784
**UCII**	**RDP sequence**	**HITChip phylotype name**	**Accession**
	Firmicutes; Clostridia; Clostridiales; unclassified Clostridiales		
	uncultured bacterium; C583	uncultured bacterium C583	AY916338
	uncultured bacterium; D191	uncultured bacterium D191	AY916361
	uncultured bacterium; K342	uncultured bacterium K342	AY916195
	uncultured bacterium; LQ86	uncultured bacterium LQ86	AY916269
	uncultured bacterium; MH87	uncultured bacterium MH87	AY916298
	uncultured bacterium; MM92	uncultured bacterium MM92	AY916304
	uncultured bacterium; M501	uncultured bacterium M501	AY916160

uncultured human intestinal bacterium; JW2H12	uncultured human gut bacterium JW2H12	AB080880
uncultured human intestinal bacterium; JW1H11	uncultured human gut bacterium JW1H11	AB080881
uncultured bacterium; OLDB-F4	uncultured bacterium OLDB-F4	AB099777
uncultured bacterium; OLDB-H1	uncultured bacterium OLDB-H1	AB099779
uncultured bacterium; M403	uncultured bacterium M403	AY916155
uncultured bacterium; Eldhufec328	Uncultured bacterium clone Eldhufec328	AY920203
uncultured bacterium; Eldhufec329	Uncultured bacterium clone Eldhufec329	AY920204
uncultured bacterium; sd56	Uncultured bacterium clone Eldhufec333	EU201635
uncultured bacterium; Eldhufec334	Uncultured bacterium clone Eldhufec334	AY920209
uncultured bacterium; Eldhufec336	Uncultured bacterium clone Eldhufec336	AY920211
uncultured bacterium; C655	uncultured bacterium C655	AY916341
uncultured bacterium; cadhufec17f05sav	uncultured bacterium cadhufec17f05	AF530343
uncultured bacterium; cadhufec18c08sav	uncultured bacterium cadhufec18c08	AF530351
Firmicutes; unclassified Firmicutes		
uncultured bacterium; OLDC-A2	uncultured bacterium OLDC-A2	AB099780
uncultured bacterium; Eldhufec332	Uncultured bacterium clone Eldhufec332	AY920207
unclassified Bacteria		
uncultured bacterium; 128g07	uncultured bacterium C736	AJ812148
uncultured bacterium; OLDB-C2	uncultured bacterium OLDB-C2	AB099778
uncultured human intestinal bacterium; JW1B2	uncultured human gut bacterium JW1B2	AB080879
uncultured bacterium from human colonic sample; HuCA6	uncultured bacterium HuCA6	AJ408962

Supplementary Table 3 Phylogenetic characterization of the UCI and UCII groups

Ribosomal Database Project (RDP)[4] alignment for the 16S rRNA target sequences of Uncultured Clostridiales I and II (UCI and UCII) detected with the HITChip microarray (October 2013). The RDP match score is 1 for all HITChip phylotypes except the UCII Uncultured bacterium clone Eldhufec333 (0.963). All sequences belong to the domain Bacteria. The RDP sequences are grouped according to their Phylum, Class, Order, Family and Genus; these are indicated up to the highest accessible taxonomic level, separated by semicolon.

Health status	Bimodal group	Enriched state	Compromised (%)	Controls (%)	FDR (%)
Severe obesity (*n*=136)	UCI	Low abundance	29	55	<0.1
Severe obesity	UCII	Low abundance	38	61	<0.1
IBS (*n*=106)	UCII	Low abundance	50	61	1
MetS (*n*=66)	*B. fragilis* group	High abundance	89	78	<0.1
MetS	*Prevotella* group	Low abundance	11	22	11
MetS	*Dialister* spp.	High abundance	36	28	13

Supplementary Table 4 Associations between health status and the bimodal taxa The column ('Enriched state') indicates the state associated with the disease. The fourth ('Compromised') and fifth ('Controls') columns show the proportion of subjects in the high-abundance state in the compromised and the healthy controls, respectively. To control for differences in subject characteristics or sample treatment between the two groups, we estimated the significance based on multiple logistic regression corrected for age, sex, body-mass index, and DNA extraction method using the log-ratio test followed by Benjamini-Hochberg correction. The associations with FDR<20% are shown.

Supplementary Notes

Supplementary Note 1 Phylogenetic analysis of the Uncultured Clostridiales I-II For the uncultured UCI and UCII groups that do not include any cultured representatives we performed a 16S rRNA target gene sequences alignment against the RDP database[4] to update their phylogenetic assignments. The majority (19/30) of the phylotypes within the UCI group were identified as members of family Ruminococcaceae, and 12 could be further assigned to genus *Acetivibrio* (Supplementary Table 3). Uncultured bacterium clones Eldhufec308 and Eldhufec312 exhibited clear bimodality (Supplementary Fig. 4). Similar abundance distributions characterized also the phylotypes within the UCII group, which could not be identified further down than order Clostridiales.

Supplementary Note 2 Cross-hybridization control Cross-hybridization can reduce the accuracy of observations in microarray analyses. We controlled this based on pre-calculated cross-alignment tables between the taxonomic groups targeted by the HITChip microarray. Cross-hybridization was negligible (<10%) between the bimodal groups and other taxa. The *B. fragilis* group, targeted by 40 probes, was an exception with 43% of shared probes with the *B. ovatus* group. Since this could potentially contribute to the bimodality of the *B. fragilis* abundance distribution, we investigated the 16 probes that were specific for the *B. fragilis* group. Bimodal abundance patterns were detected in 25% of the unique probes, suggesting that this group may contain both bimodal and smoothly varying higher-level phylotypes. The highly correlated *P. oralis* and *P. melaninogenica* groups had 13-26% shared probes. The correlation between these two groups remained high (0.81) after excluding the shared probes, however, confirming positive association. To avoid potential biases associated with different DNA extraction methods, the correlations between phylogenetic groups were calculated based on the 401 samples analysed with the mechanical lysis (see Methods).

Supplementary Note 3 HITChip phylogeny The HITChip phylogeny is binned at three taxonomic levels based on 16S sequence similarity, roughly corresponding to species/phylotype (98% 16S sequence similarity; *n*=1033) and genus (90%; *n*=130) levels, that are further classified into 23 higher-level groups including 10 phyla. In the present work we primarily focus on the genus level, which is less prone to cross-hybridization between closely related targets than the higher-resolution phylotype-level analysis.

Supplementary Note 4 Community diversity We quantified the overall community diversity by the Shannon index of the probe-level HITChip data (3631 probes targeting 1033 phylotypes) within the subset of 255 samples that were also analyzed for metagenomic richness (gene count)[2].

Supplementary Note 5 Bimodality significance As the standard Potential Analysis approach does not provide p-values for the observed bimodal patterns, we derived empirical pseudo-p-values for each taxon as the fraction of bootstrap samples that did not support the bimodality to obtain the following estimates of false discovery rates[5]: *Prevotella* <2%; *Dialister* spp., UCI and UCII 10%; and *B. fragilis* 25%.

Supplementary Note 6 DNA extraction bias When all samples with varying DNA extraction methods were included, we observed bimodal patterns also in bifidobacteria and ruminococci that thus appear to be confounded by different extraction methods, highlighting the necessity to take the extraction method into account in meta-analyses that integrate data across independent studies.

Supplementary Note 7 Kaplan-Meier analysis This is an approximation, however, as the changes in bacterial abundance are continuous and reversible unlike the life/death processes, and natural continuous fluctuations in bacterial abundance may induce some observed state shifts.

Supplementary Note 8 Community-level analysis A variety of methodologies have been suggested to detect community-level multimodality in microbiota profiling data. Such analysis can be sensitive to the choice of dissimilarity measure, clustering method, and the approach for validating the number of clusters[6]. Popular dissimilarity measures include Jensen-Shannon divergence, beta diversity measures, and correlation analyses. Partition Around Medoids (PAM) has been frequently used as a clustering method, and common methodologies for cluster number validation include the Kalinski-Harabasz, Silhouette width, and the Prediction Strength Index[6,7].

Supplementary References

1. Scheffer M. *Critical Transitions in Nature and Society.* Princeton University Press, New Jersey, United States (2009).

2. Chatelier E.L. *et al.* Richness of human gut microbiome correlates with metabolic markers. *Nature* **500**, 541-546 (2013).

3. Rajilić-Stojanović M. *et al*. Development and application of the human intestinal tract chip, a phylogenetic microarray: analysis of universally conserved phylotypes in the abundant microbiota of young and elderly adults. *Environ. Microbiol.* **11**, 1736-1751 (2009).

4. Cole, J.R. *et al.* The Ribosomal Database Project: improved alignments and new tools for rRNA analysis. *Nucleic Acids Res.* **37**, D141 (2009).

5. Storey J.D., Tibshirani R. Statistical significance for genome-wide studies. *Proc. Nat'l. Acad. Sci. USA* **100,** 9440-9445 (2003).

6. Koren, O. *et al.* A guide to enterotypes across the human body: meta-analysis of microbial community structures in human microbiome datasets. *PLoS Comp. Biol.* **9**, e1006863 (2013).

7. Tibshirani R., Walter G. Cluster validation by Prediction Strength. *J. Comp. Graph. Stat.* **14**, 511-528 (2005).
